# Supplementary material for: Variable Expressivity of the Beckwith-Wiedemann Syndrome in Four Pedigrees Segregating Loss-of-Function Variants of CDKN1C
Source: Genes (Basel). 2021 May 9;12(5):706. doi: 10.3390/genes12050706 (PMC8151838; doi:10.3390/genes12050706)
Supplement: Supplementary file 1 [file genes-12-00706-s001.zip › Supplementary Files/Table S2.pdf]

Table S2. Primer pairs and PCR conditions used for *CDKN1C* amplification

Primer pairs [7]	Amplicone	Touchdown	Program					
			iD	D	A	E	Cycles	fE
5′-CGTTCCACAGGCCAAGTGCG-3′<br>5′-GCTGGTGCGCACTAGTACTG-3′	373 bp	-	95°C<br>5min	95°C<br>45sec	62°C<br>45sec	72°C<br>45sec	35	72°C<br>5min
5′-CGTCCCTCCGCAGCACATCC-3′<br>5′-CCTGCACCGTCTCGCGGTAG-3′	278 bp	-	95°C<br>5min	95°C<br>45sec	60°C<br>45sec	72°C<br>45sec	35	72°C<br>5min
5′-ACGCCTGCAGTGGACCGAAGTGGA-3′<br>5′-GGAACCCAGCGAGGCCCCCGA-3′	688 bp	+	95°C<br>5min	95°C<br>45sec	70°C<br>45sec	72°C<br>45sec	5	72°C<br>5min
				95°C<br>45sec	68°C<br>45sec	72°C<br>45sec	10	
				95°C<br>45sec	64°C<br>45sec	72°C<br>45sec	20	
5′-CCCTCTCCCGGCCCCCTCTCG-3′<br>5′-CGCCGCCGGTTGCTGCTACATG-3′	391bp	+	95°C<br>5min	95°C<br>45sec	70°C<br>45sec	72°C<br>45sec	5	72°C<br>5min
				95°C<br>45sec	68°C<br>45sec	72°C<br>45sec	10	
				95°C<br>45sec	64°C<br>45sec	72°C<br>45sec	20	

iD: initial Denaturation; D: Denaturation; A: Annealing; E: Extention; fE: final Extention
