# Supplementary material for: Variable Expressivity of the Beckwith-Wiedemann Syndrome in Four Pedigrees Segregating Loss-of-Function Variants of CDKN1C
Source: Genes (Basel). 2021 May 9;12(5):706. doi: 10.3390/genes12050706 (PMC8151838; doi:10.3390/genes12050706)
Supplement: Supplementary file 1 [file genes-12-00706-s001.zip › Supplementary Files/Table S1.pdf]

Table S1. Consensus scoring system of Beckwith-Wiedemann syndrome

Clinical Features	Points (cut-off ≥ 4 for clinical diagnosis of BWS patients [9])	Points (cut-off ≥ 3 for molecular testing of suspected prenatal BWS cases [12])
Macroglossia	2	3
Exomphalos	2	3
Lateralized overgrowth	2	-
Multifocal and/or bilateral Wilms tumour or nephroblastomatosis	2	-
Hyperinsulinism lasting >1week	2	-
Adrenal cortex cytomegaly, placental mesenchymal dysplasia or pancreatic adenomatosis	2	3*
Birthweight >2 SDS above the mean	1	2
Facial naevus simplex	1	-
Polyhydramnios and/or placentomegaly	1	2 points each
Ear creases and/or pits	1	-
Transient hypoglycaemia (<1week)	1	-
Typical BWS spectrum tumours	1	3
Nephromegaly and/or hepatomegaly	1	2
Umbilical hernia and/or diastasis recti	1	-
Family history of inheritable BWS	-	3
Nephroureteral anomalies	-	1
Elevated hCG/α-FP/PAPP-A	-	1
Monozygotic twinning	-	1
Pregnancy from ART	-	1

*: For placental mesenchymal dysplasia only.
