# Supplementary material for: Variable Expressivity of the Beckwith-Wiedemann Syndrome in Four Pedigrees Segregating Loss-of-Function Variants of CDKN1C
Source: Genes (Basel). 2021 May 9;12(5):706. doi: 10.3390/genes12050706 (PMC8151838; doi:10.3390/genes12050706)
Supplement: Supplementary file 1 [file genes-12-00706-s001.zip › Supplementary Files/Figure S1.pdf]

atg.tcc.gac.gcg.tcc.ctc.cgc.agc.aca.tcc.acg.atg.gag.cgt.ctt.gtc.gcc.cgt.ggg.acc 20
ttc.cca.gta.cta.gtg.cgc.acc.agc.gcc.tgc.cgc.agc.ctc.ttc.ggg.ccg.gtg.gac.cac.**gag** 40
gag.ctg.agc.cgc.**gag**.ctg.**cag**.gcc.cgc.ctg.gcc.gag.ctg.aac.gcc.gag.gac.cag.aac.cgc 60
tgg.gat.tac.gac.ttc.**cag**.cag.gac.atg.ccg.ctg.cgg.ggc.cct.gga.cgc.ctg.**cag**.tgg.acc 80
gaa.gtg.gac.agc.gac.tcg.gtg.ccc.gcg.ttc.tac.cgc.gag.acg.gtg.cag.gtg.ggg.cgc.tgc 100
cgc.ctg.ctg.ctg.gcg.ccg.cgg.ccc.gtc.gcg.gtc.gcg.gtg.gct.gtc.agc.ccg.ccc.ctc.gag 120
ccg.gcc.gct.gag.tcc.ctc.gac.ggc.ctc.**gag**.gag.gcg.ccg.gag.cag.ctg.cct.agt.gtc.ccg 140
gtc.ccg.gcc.ccg.gcg.tcc.acc.ccg.ccc.cca.gtc.ccg.gtc.ctg.gct.cca.gcc.ccg.gcc.ccg 160
gct.ccg.gct.ccg.gtc.gcg.gct.ccg.gtc.gcg.gct.ccg.gtc.gcg.gtc.gcg.gtc.ctg.gcc.ccg 180
gcc.ccg.gcc.ccg.gct.ccg.gct.ccg.gct.ccg.gcc.ccg.gct.cca.gtc.gcg.gcc.ccg.gcc.cca 200
gcc.ccg.gcc.ccg.gcc.ccg.gcc.ccg.gcc.ccc.gcc.ccg.gcc.ccg.gcc.ccg.gac.gcg.gcg.cct 220
caa.gag.agc.gcc.**gag.cag**.ggc.gcg.aac.**cag**.ggg.**cag**.cgc.ggc.**cag.gag**.cct.ctc.gct.gac 240
cag.ctg.cac.**tcg**.ggg.att.**tcg**.gga.cgt.ccc.gcg.gcc.ggc.acc.gcg.gcc.gcc.agc.gcc.aac 260
ggc.gcg.gcg.atc.aag.aag.ctg.tcc.ggg.cct.ctg.atc.tcc.gat.ttc.ttc.gcc.aag.cgc.aag 280
aga.tca.gcg.cct.gag.aag.tcg.tcg.ggc.gat.gtc.ccc.gcg.ccg.tgt.ccc.tct.cca.agc.gcc 300
gcc.cct.ggc.gtg.ggc.tcg.gtg.gag.cag.acc.ccg.cgc.aag.agg.ctg.cgg.tga

Figure S1. Coding sequence of the *CDKN1C* gene. Sense codons that can be mutated in stop codons by single nucleotide substitutions are highlighted in yellow. Sense codons mutated in nonsense codons in BWS-associated *CDKN1C* mutations are in bold. The natural stop codon TGA is in grey. Codons of the Kinase inhibitor domain are underlined in orange; in pink those of the PAPA domain; in blue those of the PCNA binding domain. The starting codon of isoform A is highlighted in green; the starting codon of isoform B is highlighted in light blue.
